# MICU1 may be a promising intervention target for gut-derived sepsis induced by intra-abdominal hypertension

**DOI:** 10.1038/cddiscovery.2016.80

**Published:** 2016-11-28

**Authors:** Yuxin Leng, Qinggang Ge, Zhiling Zhao, Kun Wang, Gaiqi Yao

**Affiliations:** 1 Department of Intensive Care Unit, Peking University Third Hospital, North Garden Road, No. 49, Haidian District, Beijing 100191, People’s Republic of China

## Abstract

Intra-abdominal hypertension (IAH) is a common and serious complication in critically ill patients, for which there is no targeted therapy. IAH-induced dysfunction of intestinal barriers is closely associated with oxidative imbalances, which are considered to provide a pathophysiological basis for subsequent gut-derived sepsis. However, the upstream mechanism that produces oxidative damage during IAH remains unknown. It is not clear whether ‘mitochondrial Ca^2+^ uptake 1’ (MICU1, the key protein regulating the oxidative process) is involved in preventing Ca^2+^_m_ (mitochondrial Ca^2+^) overload. Here, we detected changes in the expression of MICU1 during the development of increased intestinal permeability in rats with IAH, and we explored the related mechanism regulating epithelial-barrier functions by knocking-down *micu1* in Caco-2 cells. Our results demonstrated that, to combat IAH-induced dysfunction of intestinal barriers, MICU1 undergoes a compensatory increase in expression, whereas ‘mitochondrial calcium uniporter’ (MCU) – a conserved Ca^2+^ transporter – becomes transcriptionally suppressed. Silencing the expression of MICU1 destroyed Caco-2 cell barrier integrity, promoted paracellular permeability, and impaired the expression of tight junction proteins (occludin, ZO-1, and claudin 1). Meanwhile, oxidative imbalances were induced; malondialdehyde (MDA), a product of oxidation, was increased and antioxidant products (GSH-Px, CAT, and SOD) were decreased. In MICU1-deficient Caco-2 cells, proliferation was inhibited and apoptosis was promoted. Collectively, our results indicate that MICU1-related oxidation/antioxidation disequilibrium is strongly involved in IAH-induced damage to intestinal barriers. MICU1-targeted treatment may hold promise for preventing the progression of IAH to gut-derived sepsis.

## Introduction

Intra-abdominal hypertension (IAH) is a sustained or repeated increase in intra-abdominal pressure (IAP), greater than or equal to 12 mmHg (10 mmHg for children), without well-defined intervention strategies.^1^ Intestinal barrier functions have been confirmed to be significantly damaged during IAH, which may trigger gut-derived sepsis and multi-organ failure (MOF).^2–5^ Oxidative stress following IAH-induced ischemia has been implicated as the mechanism underlying the damage. It has been reported that the elevation in IAP causes disturbances in the intestinal microcirculation, which are followed by enhanced oxidant responses and bacterial translocation.^5–8^ The balance between oxidative stress and antioxidation processes can be destroyed even before the level of IAP matches the level in the diagnostic criteria.^9^ However, the upstream mechanism underlying the oxidative imbalance induced by increased intestinal permeability remains unclear.

Mitochondrial Ca^2+^ (Ca^2+^_m_) homeostasis is critical for maintaining normal cellular physiology. Under normal conditions, the Ca^2+^ concentration in the mitochondrial matrix is maintained 5–6 orders of magnitude lower than its equilibrium level. ‘Mitochondrial Ca^2+^ uptake 1’ (MICU1), which is an EF hand Ca^2+^ binding protein, monitors mitochondrial Ca^2+^ levels and sets a Ca^2+^ threshold for Ca^2+^_m_ uptake, mediated by ‘mitochondrial calcium uniporter’ (MCU). Therefore, MICU1 prevents Ca^2+^m overload and the associated oxidative cellular damage.^10,11^ The regulatory function of MICU1, namely preventing oxidative damage, has been confirmed in HeLa cells, hepatocytes, and endothelial cells. It has been reported that, in the absence of MICU1, excessive MCU-mediated Ca^2+^ uptake leads to overproduction of mitochondrial reactive oxygen species (mROS) and sensitivity to apoptotic stimuli.^10–12^

Accordingly, considering the close connections between MICU1, oxidative stress, and IAH-induced intestinal barrier dysfunction, it is reasonable to hypothesize that dysfunction of MICU1 is the upstream mechanism for IAH-related mucosal barrier damage. In the present study, we aimed to investigate the IAH-induced changes in the expression of MICU1 and MCU in rats. In addition, we tried to verify the importance of MICU1 in maintaining the integrity of the intestinal epithelium, using genetic knockdown.

## Results

### A 90-min exposure to IAH elicited increased intestinal permeability

**Figure 1:**
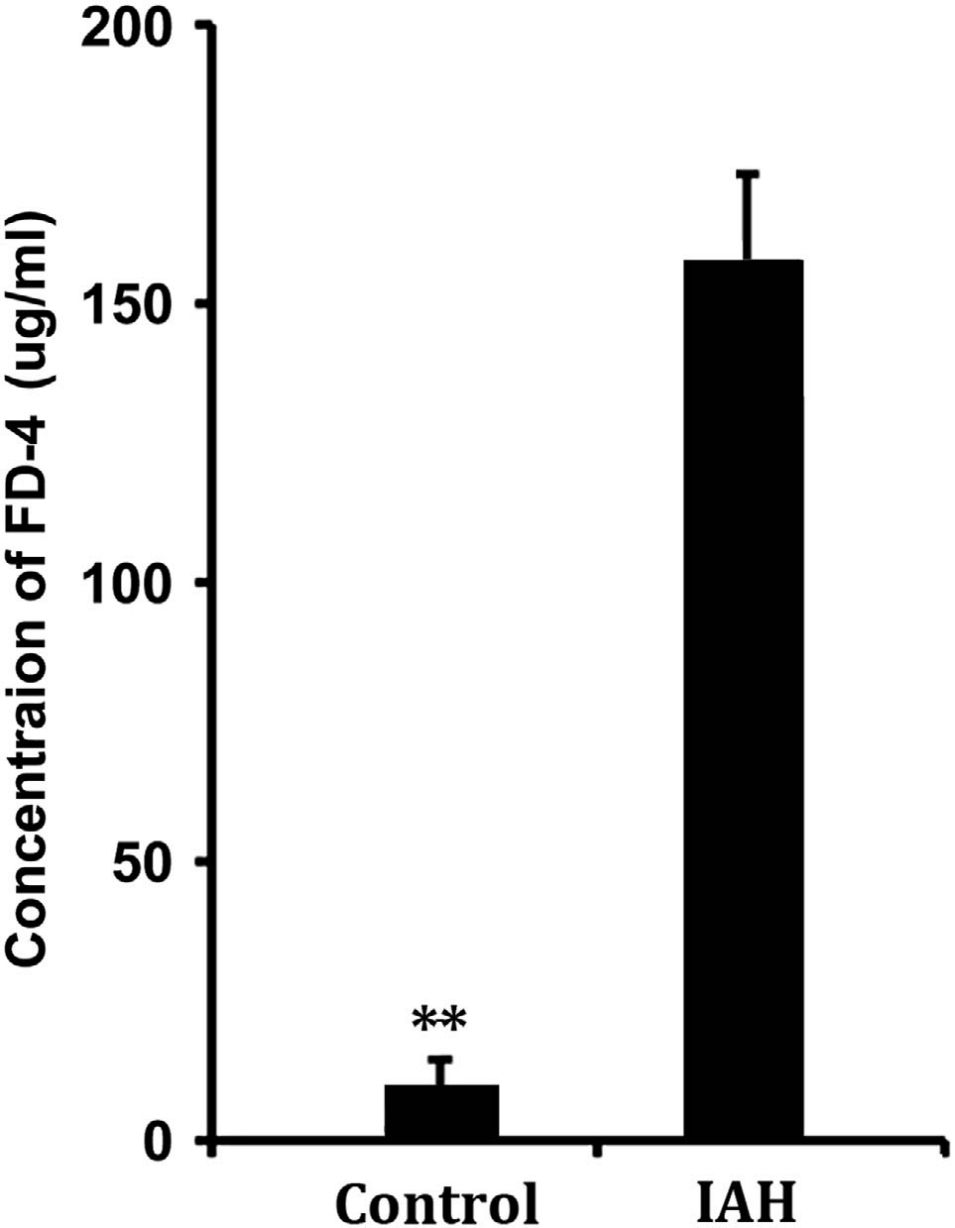
Establishment of IAH-induced increased intestinal permeability in rats. Ninety minutes exposure to an IAP of 20 mmHg was determined to successfully induce increased intestinal permeability models. The concentration of FD-4 in portal blood detected was significantly higher than that of control rats. The data are shown as mean±S.D. values and compared by independent *t*-test **P<0.01.

We determined gut permeability to macromolecules by measuring FD-4 leakage from the gut cavity into the portal circulation. As shown in Figure 1, similar to our previous findings, a 90-min exposure to IAH resulted in a significant increase in the amount of FD-4 in the plasma of portal blood (control *versus* IAH: 11.1±5.9 μg/ml *versus* 158.1±16.7 μg/ml), indicating that IAH-induced increased permeability models had been successfully established.

### Abnormal gene expression profiles were detected for MICU1 and MCU in rats with IAH

**Figure 2:**
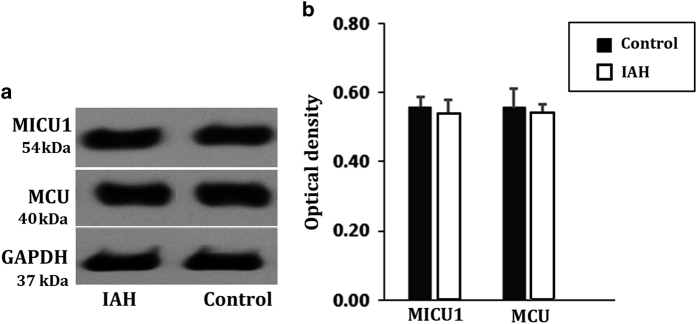
Expression analysis on colonic MICU1/MCU at protein level by western blotting in rats with IAH 90-min.The exposure to an IAP of 20 mmHg had no effect on MICU1 and MCU protein expressions. No significant differences were found between control group and IAH group. (**a**) The band of western blotting. (**b**) Analysis on gray value. The data are shown as mean±S.D. values and compared by independent *t*-test.

No significant changes in the levels of MICU1 and MCU were detected in the rats with acute IAH, using western blotting and qRT-PCR (Figure 2; Table 1). However, changes in the levels of MICU1 and MCU were determined at the transcriptional level (Table 1). MICU1 was significantly upregulated, whereas MCU was downregulated (Table 1). This imbalance between MICU1 and MCU, at mRNA level, during a 90 min exposure to IAH might be the result of an attempt by the cells to minimize IAH-induced damage to the intestinal barrier. Therefore, the compensatory increase in MICU1 expression suggested its importance in intestinal barrier protection.

### Knockdown of MICU1 destroys the epithelial-barrier function in cultured Caco-2 cells

**Figure 3:**
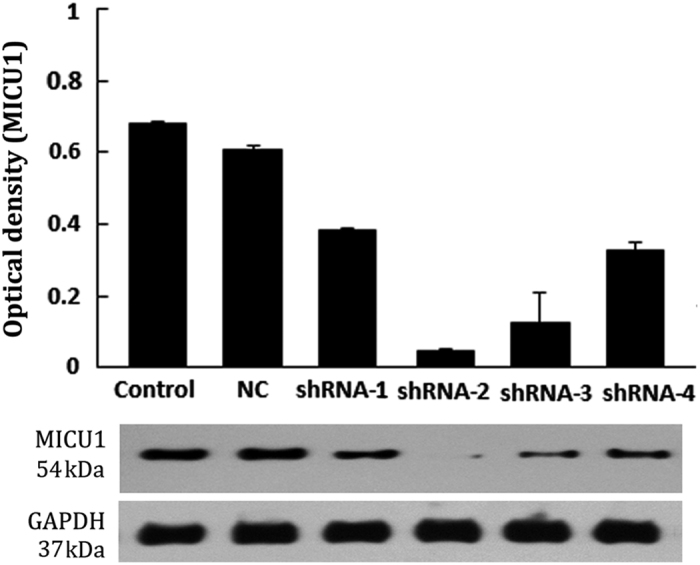
Identification of silencing gene *micu1* in Caco-2. MICU1-knockdown was identified by western blotting analysis. The four distinct hairpins (as shown in Table 2) gave 44.1, 92.6, 82.4 and 51.5% MICU1 protein knockdown, respectively. NC (negative control group), Caco-2 cells transfected with empty lentiviral vector.

To elucidate the function of MICU1, we silenced its expression using four distinct RNA hairpins targeting the gene (Table 2). Notably, shRNA-2 was the most effective, resulting in ~92.6% knockdown of MICU1 protein expression (Figure 3). All of the following results are from knockdown studies performed using shRNA-2.

**Figure 4:**
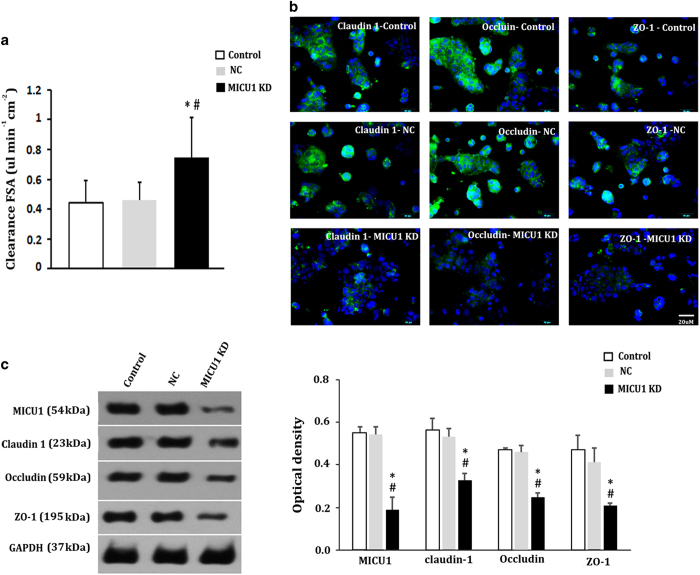
Involvement of MICU1 in intestinal barrier integrity *in vitro*. (**a**) Clearance of FSA. (**b**) Immunofluorescence analysis on tight junction proteins, claudin 1, occludin, and ZO-1 (green). (**c**) Western blotting analysis of tight junction proteins. Results are shown as mean±S.D. of three independent experiments. Statistical analysis was performed by one-way ANOVA test and independent *t*-test. *versus* control, *P<0.05;* versus*NC, ^#^*P*<0.05. NC (negative control group), Caco-2 cells transfected with empty lentiviral vector. MICU1-KD, MICU1-knockdown.

Examination of tight junction proteins, the clearance of fluorescein sodium salt (FSA), and tansepithelial electrical resistance (TEER) studies demonstrated that MICU1 is essential for the protection of the mechanical barrier function of intestinal tissue *in vitro* (Figure 4). Formation of a Caco-2 cell monolayer was initially validated by TEER measurements. For the control Caco-2 cells, and those transfected with an empty lentiviral vector (negative shRNA control group, NC), the TEER values detected were all higher than 250 Ωcm^2^ at 14 days, indicating perfect compactness and integrity of their monolayers. Using these cellular monolayers, we noticed that the clearance of FSA in the MICU1-deficient group was significantly higher than that of the control group and the NC group (Figure 4a), whereas the clearance of FSA was equivalent in the control group and the NC group. The expression results for the TJs further confirmed the indispensability of MICU1 in maintaining the integrity of intestinal barriers. As shown in Figures 4b and c, both the immunofluorescence and the western blotting analyses showed that the levels of claudin 1, occludin, and ZO-1 – which are responsible for TJ permeability and paracellular transport – were all markedly reduced when MICU1 was silenced.

### MICU1 dysfunction is the upstream mechanism underlying the oxidative disturbance that damages epithelial barriers during IAH

**Figure 5:**
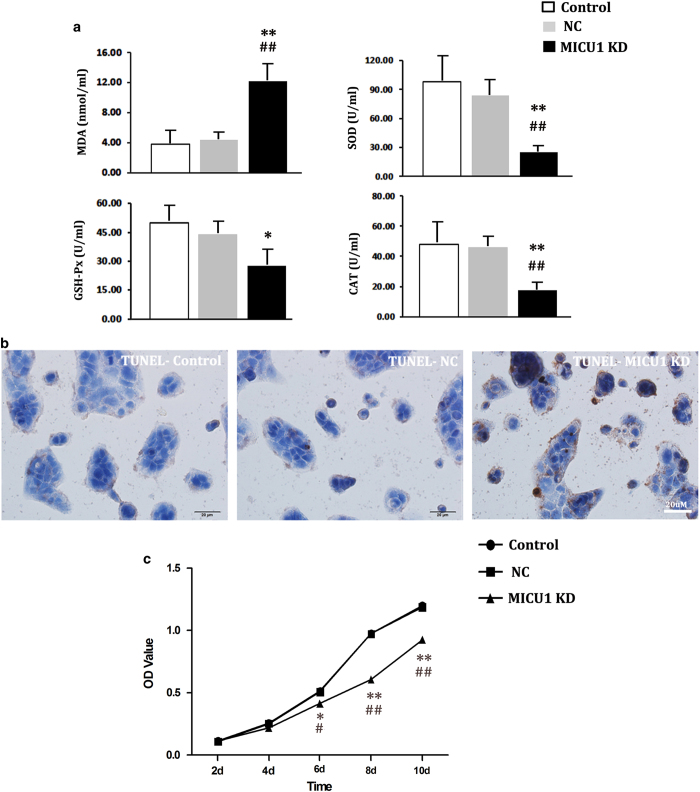
MICU1 regulates the oxidative responses, apoptosis and cell growth *in vitro*. (**a**) Detection of oxidative imbalance. In MICU1-KD cells, the oxidative product MDA was significantly increased, whereas the antioxidant products (GSH-Px, CAT, and SOD) decreased. (**b**) TUNEL stain. The nuclei of the apoptotic cells were positively stained brownish yellow. The proportion of the positively immunostained cells were significantly higher than those of NC group and control group. (**c**) Growth curve. From day 6, the OD values of cultured MICU1-KD cells were significantly lower than those of NC and control. Statistical analysis was performed by one-way ANOVA test and independent *t*-test. *versus* control, *P<0.05, **P<0.01; *versus* NC, ^#^*P*<0.05, ^##^*P*<0.01. NC (negative control group), Caco-2 cells transfected with empty lentiviral vector. MICU1-KD, MICU1-knockdown.

The results of mechanistic studies have shown that MICU1 deficiency led to oxidative imbalances, which are closely associated with the damage to intestinal mucosa that can be induced under acute, slightly increased IAP.^9^ Furthermore, these studies have shown that MICU1 deficiency inhibited the growth of Caco-2 cells and sensitized them towards apoptotic cell death. As shown in Figure 5a, the oxidative balance became significantly disturbed in the current study, as demonstrated by increased levels of MDA – a lipid peroxidation product – and decreased antioxidant products (GSH-Px, CAT, and SOD). Meanwhile, the TUNEL assay demonstrated prominent apoptosis of colonic epithelial cells, resulting from the knockdown of MICU1 (Figure 5b). The rate of apoptosis was significantly increased on MICU1-knockdown (MICU1-KD *versus* control *versus* NC: 33.16±10.01% *versus* 3.64±1.33% *versus* 4.18±1.68%; *P*<0.05). In addition, the MTT colorimetric assay demonstrated that the viability of Caco-2 cells was significantly affected when MICU1 was silenced. Across the 10-day culturing period, the OD values for the culture of MICU1-KD colonic epithelial cells were significantly lower than those of the control group or the NC group (Figure 5c); the OD values of the control group and the NC group matched each other.

## Discussion

Damage to the barrier functions of the gastrointestinal tract, triggered by IAH-associated ischemia and subsequent oxidative injury, is considered the pathophysiological basis for the IAH-associated MOF cascade.^9,13^ In this study, we further explored the upstream mechanisms underlying IAH-induced increased intestinal permeability. We found that MICU1, the gatekeeper of mitochondrial calcium ion influx, is deeply involved in the regulation of oxidative damage, by modulating the proliferation and apoptosis of colonic epithelial cells. Silencing of MICU1 destroyed Caco-2 cell barrier integrity, promoted paracellular permeability, and impaired the expression of TJ proteins. All of these alterations can be found in rats with IAH.^9^ In line with the findings in cultured cells, a compensatory increase in MICU1 was detected in rats with IAH. All of these results suggest that MICU1 is essential for regulating intestinal barrier function. We conclude that disturbances in MICU1 levels are involved in the increased intestinal permeability induced by IAH.

MICU1 is also called CBARA1 (calcium binding atopy-related autoantigen 1). It has recently be demonstrated that it functions as a gatekeeper that sets the Ca^2+^ threshold for Ca^2+^ uptake by MCU, to regulate oxidative stress, both *in vivo* and *in vitro*.^10,12,14^ As this gatekeeper function was identified, a considerable number of studies investigated the expression and function of MICU1 in undifferentiated human embryonic stem cells (hESCs), hepatocytes, dermal endothelial cells, HeLa cells, breast epithelia, and pancreatic β-cells, and other cell types.^15–21^ Nevertheless, before our investigations, few studies had focused on the expression of MICU1 in enterocytes or its involvement in IAH-induced intestinal barrier damage. In fact, mitochondrial dysfunction has been confirmed in the development of IAH, surgical stress, and other similar conditions. Exposure to increased IAP is reported to lead to mitochondrial swelling and discontinuous intercellular tight junctions.^3^ Surgical stress, the most common cause for IAH, results in alterations to mitochondrial respiration and the thiol redox status of enterocytes. These changes were associated with altered mitochondrial matrix enzyme activity, decreased superoxide dismutase activity, induction of the mitochondrial permeability transition, as well as impairments in the Ca^2+^_m_ flux.^22^ Meanwhile, previous studies have elucidated that intestinal oxidative stress is responsible for the altered intestinal permeability seen during surgical stress, or the development of IAH.^9,23^ Thus, it is not difficult to understand how the intestinal barrier dysfunction that develops under IAH, or surgical stress, is related to disturbances in MICU1 levels. This is because MICU1 is involved in oxidative regulation, as we have confirmed in our studies.

MICU1 has been determined to be vital for liver tissue repair and hESC self-renewal and pluripotency.^15,16^ Antony *et al.* found that on liver-specific MICU1 loss, partial hepatectomy resulted in lethargic mice. Furthermore, the pro-inflammatory phase does not resolve in such mice and liver regeneration fails, with evidence of impaired cell cycle entry and extensive necrosis. Preventing the opening of the mitochondrial permeability transition pore (mPTP) in MICU1-knockdown mice successfully rescued hepatocyte proliferation.^15^ Moreover, MICU1-knockdown in hESCs attenuated cell growth and produced G_0_/G_1_-phase cell cycle arrest, indicating that MICU1 has a role in maintaining stemness, cell cycle progression, and proliferation.^18^ Our results are consistent with these findings. As shown in Table 1, MICU1 struggled to antagonize the oxidative stress, induced by a 90-min exposure to IAH, by increasing its transcriptional expression. It should be noted, however, that no obvious changes were detected in the levels of MICU1. Studies with *micu1* knocked-down enterocytes further demonstrated that MICU1 protects tissues by regulating oxidative stress and cellular proliferation. As shown in Figure 5, without *micu1*, the apoptotic rate of Caco-2 cells increased, whereas cellular viability was lowered; this was accompanied by an oxidative imbalance. All of these changes were responsible for the disrupted TJ permeability and paracellular transport.

The gut is the most sensitive to increased IAP. We previously reported that even mild increase in IAP (8–12 mmHg) is accompanied by slight mucosal damage and an oxidative imbalance.^9^ Our findings have been supported by the work of Maddison *et al*.^24^ We found that as the IAP increases to 20 mm Hg for 90 min, intestinal barriers become seriously disrupted and dysbiosis occurs.^25^ However, in the present study, at the same level of IAH, MICU1 exhibited a compensatory response towards the increased intestinal permeability, indicating that under IAH, the gut promotes a self-protective mechanism. Further studies would be needed to establish whether MICU1 would be disrupted as the course/degree of IAH progresses, and to determine what roles are played by MICU1 in the subsequent abdominal compartment syndrome and gut-derived sepsis.

## Conclusions

The current study is the first to identify the MICU1-related upstream mechanism for IAH-induced oxidative imbalances, and the subsequent increase in the intestinal permeability. MICU1 may be a promising target for intervening in IAH-induced gut-derived sepsis and MOF. Because MICU1 has a key role in maintaining intestinal barrier integrity by regulating oxidative stress and cellular proliferation.

## Materials and Methods

### Animal studies

#### Establishment of IAH-induced increased intestinal permeability in rat models

To explore the involvement of MCU/MICU1 in IAH-induced intestinal permeability changes, 8 male SPF Sprague-Dawley rats (8-weeks-old, weight: 200–250 g) were randomly assigned to the ‘IAH group’ (20 mmHg, *n*=4), or to the control group (*n*=4). The acute IAH animal model (20 mmHg) was established using a 90-min nitrogen pneumoperitoneum procedure. Increased intestinal permeability was identified by the detection of intestinal permeability to FITC-dextran (FD-4, molecular weight: 4000 Da; Sigma-Aldrich, St. Louis, MO, USA, Product No. 68059) as previously described.^9^ Briefly, following anesthesia by intraperitoneal injection of sodium pentobarbital (40 mg/kg), nitrogen pneumoperitoneum was created by a disposable venous infusion needle connected to a micro-infusion pump. The micro-infusion pump was linked to a blood pressure meter to dynamically monitor the IAP. After 90 min, a 10-cm segment of the distal ileum with preserved superior mesenteric vessels was dissected 3 cm proximal to the cecum. One milliliter of phosphate-buffered saline (PBS; 0.1 mol/l, pH 7.2) containing 25 mg of FD-4 was injected into this ligated 10-cm segment of intestinal lumen, and the lumen was carefully replaced in the abdomen, covered, and protected with gauze soaked in warm saline. Portal venous blood samples were collected to analyze the FD-4 concentrations spectrophotometrically, after 30 min, at an excitation wavelength of 492 nm and an emission wavelength of 518 nm. The colonic tissues were frozen by immersion in liquid nitrogen and stored at −80 °C, until needed for further experiments. For the control group, nitrogen was not injected.

All experimental procedures, including the care and handling of animals, were performed following international guidelines (Guide for the Care and Use of Laboratory Animals, Institute of Laboratory Animal Resources, Commission on Life Sciences, National Research Council, National Academy Press, Washington, DC, USA, 1996).^26^ The rationale, design, and protocols for our study were approved by the Peking University Biomedical Ethics Committee-Experimental Animal Ethics Branch (Approval No. LA2013-12), before the initiation of experiments. The rats were housed solitarily in polypropylene cages and kept under standard controlled environmental conditions with 12 h light/dark cycles. The rats had free access to standard rat chow and water, which were autoclaved before use. The rats were deprived of food, but not water, for 12 h before inducing nitrogen pneumoperitoneum for 90 min (described below).

All surgeries were conducted under sodium pentobarbital anesthesia (intraperitoneal injection, 40 mg/kg) and all efforts were made to minimize pain. After the procedure was complete, the animals were euthanized using an overdose of sodium pentobarbital (intraperitoneal injection, 160 mg/kg).^9^

### Changes in the expression of MICU1/MCU in rats with IAH

#### Western blotting

Standard western blotting analyses of MICU1 and MCU were conducted as previously described.^9^ Colonic tissue samples (or cultured cells) were homogenized in lysis buffer (20 mmol/l Tris–HCl (pH 7.5), 1% Triton X-100, 0.2 mol/l NaCl, 2 mmol/l ethylenediaminetetraacetic acid, 2 mmol/l ethylene glycol tetraacetic acid, 1 mol/l dithiothreitol, and 2 mol/l aprotinin). Proteins (60 μg) were electrophoresed using SDS-PAGE (8%) and transferred to a polyvinylidene fluoride membrane. The membranes were blocked for 1 h with nonfat dried milk in Tris-buffered saline containing 0.05% Tween-20 (TTBS) at room temperature. They were then incubated overnight, with gentle shaking, at 4 °C with antibodies against MCU and MICU1. (Details for all the primary antibodies that we applied are provided in Table 3). Thereafter, goat anti-rabbit fluorescently labeled secondary antibodies (1:10 000; LI-COR Biosciences, Lincoln, NE, USA) were added for 1 h to the membranes at room temperature. Bound proteins were visualized following scanning of the membranes with an Odyssey Infrared Imaging System (LI-COR Biosciences, Lincoln, NE, USA).

#### Quantitative reverse transcription PCR analysis

Total RNA was isolated from colonic tissue (or Caco-2 cells), using TRIzol Reagent (Invitrogen, Carlsbad, CA, USA), according to the manufacturer’s instructions. RNA samples were then standardized and reverse transcribed using a Transcriptor First Strand cDNA Synthesis Kit (Roche, Basel, Switzerland) with an oligo-dT/random nonamer primer mixture. RT-qPCR was carried out using a ‘Roche FS Universal SYBR Green Master’ mixture (Roche), according to the manufacturer’s instructions, and a ‘Viia7’ system (Applied Biosystems, Shanghai, China).

Thermal cycling parameters: Started with 10 min at 95 °C, followed by 40 cycles of 95 °C for 15 s, 60 °C for 30 s, and 72 °C for 30 s. The specificity of the PCR products was verified by melting curve analysis. Specific primers for both the genes, *Mcu (NM_001106398.1, 1364 bp)* and *Micu1 (NM_199412.1, 2300 bp*) were designed by Qiyin Biotechnology (Shanghai, China). The primer sequences were:

- *Mcu* -F: 5′- ACCCTGAACGATGTGAAGA-3ʹ
- *Mcu* -R: 5ʹ- CTCCGCTTTCCTGCTAAT-3ʹ
- *Micu1*-F: 5ʹ- CAAGTCTGGCTTATGTTCG-3ʹ
- *Micu1*-R: 5ʹ- AGATTCTCCCGTCTACCG-3ʹ

## Cell studies

### Cell culture

Caco-2 cells were cultured at 37 °C under a 5% CO_2_ humidified-atmosphere in DMEM, supplemented with 10% fetal bovine serum, 1% nonessential amino acids, 100 U/ml penicillin, and 100 μg/ml streptomycin. Cells were used in experiments ~14 days after achieving confluence, to allow for their differentiation into intestinal epithelial cells. The medium was replaced every 3 days.

### Generation of stable MICU1-knockdown cell lines

We generated stable clones using lentiviral shRNAs targeting different regions of the *micu1* gene (Table 2). Four different lentiviruses, each carrying shRNAs targeting different regions of *micu1*, were produced by co-transfecting 293T cells with MICU1 lentiviral shRNA constructs (KeyGEN BioTECH, Jiangsu, China; Table 2), psPAX2, and pMD2.G (Addgene, Cambridge, MA, USA), as previously described.^10,27^ Caco-2 cells (5×10^5^ per well) grown in 6-well plates were transduced with MICU1 lentiviruses, selected with puromycin (2 μg/ml) 72 h post-transduction for 6–10 days, and then expanded. Knockdown was assessed by western blotting.

### Influence of MICU1 on epithelial-barrier function in cultured Caco-2 cells

#### TEER and detection of transport using FSA

To analyze the effect of MICU1 on paracellular permeability, a monolayer of Caco-2 cells was established and measured as TEER using a Millicell-ERS device (Millipore, Bedford, MA, USA), as previously described.^28^ Briefly, 2×10^5^ cells/ml were seeded on Transwell permeable supports and cultured. Then, TEER was measured every day and calculated as:

$$\mathrm{TEER}=\left(R_{m}-R_{i}\right)\times S$$

(*R*_m_, transmembrane resistance; *R*_i_, intrinsic resistance of a cell-free medium; and *S*, the surface area of the membrane in cm^2^, 1.12 cm^2^).

When the TEER value exceeded 250 Ωcm^2^, paracellular transport through Caco-2 cell monolayers was determined by measuring the apical-to-basolateral clearance of fluorescein (FSA, sodium salt) (Sigma-Aldrich, St Louis, MO, USA, Lot. F6377), as previously described.^29^ Briefly, once the TEER values had been determined, the medium in all compartments was replaced with Hank’s balanced salt solution; FSA was then added to the apical compartment (10 mg/l in 200 μl). One hour after the addition of FSA, 100 μl solution was taken from the basolateral compartment and the fluorescence was measured (*λ*_ex_: 428 nm; *λ*_em_: 536 nm). FSA clearance (CL_FSA_) was calculated using the equation:

CL_FSA_=*F*_ab_/(*F*_FSA_×*S*); where ‘*F*_ab_’ represents the flux of FSA in fluorescence units/h; ‘*F*_FSA_’, the fluorescence of FSA in the apical compartment; and ‘*S*’, the surface area of the membrane in cm^2^.

#### Immunofluorescence analysis of tight junction proteins (TJs)

To explore the association between the expression of MICU1 and TJs, standard immunofluorescence and western blotting were performed in Caco-2 cells (control) and MICU1-silenced cells. For immunostaining, cells were seeded onto glass coverslips in 6-well plates and fixed overnight with 4% formaldehyde. The cells were then permeabilized with 0.1% Triton X-100 in PBS and stained with anti-claudin 1, anti-occludin, or anti-ZO-1 antibodies (5×10^5^ per well). Following a PBS wash, the cells were incubated with fluorescein isothiocyanate-linked secondary antibodies, for 1 h at room temperature (KeyGEN BioTECH, Jiangsu, China, Lots. KGAA26 & KGAA27). Nuclei were counterstained with 4',6-diamidino-2-phenylindole. The sections were scanned by confocal microscopy (Olympus IX51, Tokyo, Japan).

### The influence of MICU1 on the pro-oxidant/antioxidant balance in cultured Caco-2 cells

The influence of MICU1 on the pro-oxidant/antioxidant balance was measured by ELISA. We detected the levels of MDA (Lot. KGT003), glutathione peroxidase (GSH-Px, Lot. KGT014), catalase (CAT, Lot. KGT017), and serum superoxide dismutase (SOD, Lot. KGT00150) in cultured Caco-2 cells, according to the manufacturer’s instructions (KeyGEN BioTECH, Jiangsu, China). Each test was repeated three times. Measurements were quantified by spectrophotometric recordings (Thermo Fisher Scientific, Waltham, MA, USA).

### TUNEL assay for apoptosis

Apoptosis resulting from the knockdown of MICU1 detected by a terminal deoxynucleotidyl transferase dUTP nick-end labeling kit (TUNEL, KeyGEN BioTECH, Jiangsu, China, Lot. KGA702), according to the manufacturer’s instructions. The percentage of TUNEL-positive cells was calculated, and compared between both groups. The experiment was repeated three times. Five randomly selected fields were chosen in each section. In total, 15 datasets were recorded for each group and compared.

### MTT assay for cell growth

Cultured cells were seeded at a density of 3×10^3^ cells per well in 96-well microtiter culture plates. After 2, 4, 6, 8, and 10 days of culturing, separate wells received a 20 μl addition of MTT solution (5 mg/ml in PBS; St. Louis, MO, USA); these were each incubated for a further 4 h. On termination, the supernatant was aspirated and MTT was converted to formazan by metabolically viable cells. The formazan was then dissolved in 150 μl of DMSO. The plates were mixed for 10 min on a gyratory shaker, and absorbance was measured at 490 nm.

### Statistical analysis

Statistical analyses were performed using SPSS 16.0 software (SPSS, Chicago, IL, USA). Data were expressed as mean values±S.D. One-way analysis-of-variance tests and independent *t*-tests were performed. *P*-values <0.05 were considered statistically significant.

## Figures and Tables

**Table 1:** Gene expression levels of MICU1 and MCU in rats with an IAP of 20 mmHg

*Gene symbol*	*logFC*	t	P*-value*	*adj.* P*-val*	*B*
*MICU1*	1.529	6.99	0.0009	0.0738	0.9661
*MCU*	−2.826	−1 1.91	0.0000	0.0057	2.0278

Abbreviations: logFC, a positive results indicated upregualtion; a negative results indicated downregulation; *t*, *t*-test statistic; *P*-value, unadjusted *P-*value; adj. *P*-val, adjusted *P*-value; B, expression index.

The differential expression levels were calculated using a moderate *t*-test implemented in the Bioconductor limma package (R-statistical software) at http://www.bioconductor.org.

**Table 2:** Details for micu1 gene and lentiviral shRNA sense sequences

*Gene (name, ID)*	*Ref seq*	*shRNA sense sequence (no. of starting base)*
*micu1 10367*	NM_001195519.1	CAGAGAAATTTTGAAATTGC (191)
*micu1 10367*	NM_001195519.1	GTATGCGCCACAGAGATCGT (303)
*micu1 10367*	NM_001195519.1	GAGAATTACTGAGAGGCAGT (493)
*micu1 10367*	NM_001195519.1	GACATTTCAGGAGGTGGAGA (610)

**Table 3:** Details of the first antibodies

*Reagents*	*Claudin 1*	*Occludin*	*GAPDH*	*MICU1*	*ZO-1*	*MCU*
Company	Abcam	Abcam	Abcam	Abcam	Abcam	Abcam
Product number	ab15098	ab31721	Ab8245	ab102830	ab190085	ab121499
Source	Rabbit	Rabbit	Mouse	Rabbit	Goat	Rabbit
Dilution	1:200	1:200	1:1000	1:1000	1:200	1:200

## Abbreviations

Ca_m_^2+^: mitochondrial Ca^2+^
CAT: catalase
CL_FSA_: FSA clearance
DMSO: dimethyl sulfoxide
FD-4: FITC-dextran, 4000Da
*F*ab: the flux of FSA in fluorescence units
*F*_FSA_: the fluorescence of FSA in the apical compartment
FSA: fluorescein sodium salt
GSH-Px: glutathione peroxidase
hESCs: human embryonic stem cells
IAP: intra-abdominal pressure
IAH: intra-abdominal hypertension
KD: knockdown
MCU: mitochondrial calcium uniporter
MDA: malondialdehyde
MICU1: mitochondrial Ca^2+^ uptake 1
MOF: multi-organ failure
mPTP: mitochondrial permeability transition pore
mROS: mitochondrial reactive oxygen species
MTT: 3-(4,5)-dimethylthiahiazo (-z-y1)-3,5-di- phenytetrazoliumromide
NC: negative shRNA control
SOD: serum superoxide dismutase
TEER: transepithelial electrical resistance
TTBS: Tris-buffered saline containing Tween-20
TJ: tight junction
TUNEL: terminal deoxynucleotidyl transferase dUTP nick-end labeling.
